# Drug prescriptions in elderly hospitalized patients with cognitive impairment in the Italian Dementia Friendly Hospital project

**DOI:** 10.3389/fphar.2024.1474986

**Published:** 2024-11-12

**Authors:** Stefano Govoni, Alessia Rosi, Stefania Preda, Cristina Lanni, Stefano Cappa, Nicola Allegri

**Affiliations:** ^1^ Department of Drug Sciences, University of Pavia, Pavia, Italy; ^2^ CEFAT (Center of Pharmaceuticals Economics and Medical Technologies Evaluation), University of Pavia and S.A.V.E, Milano, Italy; ^3^ Department of Brain and Behavioral Sciences, University of Pavia, Pavia, Italy; ^4^ IRCCSC Mondino, Pavia, Italy; ^5^ Institute of Advanced University Education-IUSS, Pavia, Italy

**Keywords:** aging, cognitive impairment, drug appropriateness, sedative load, anticholinergic load, drug interactions

## Abstract

**Objective:**

The aim of the study was to characterize drug prescription patterns in elderly patients hospitalized in acute wards as a function of cognitive status and staff training.

**Methods:**

We recorded clinical parameters reflecting health status and drug prescriptions at admission, during hospital stay, and at discharge before and after a short staff training on the needs of aged cognitively impaired patients. Participants aged 65 and older had a Mini-Mental State Examination (MMSE) score ≥16. The number of prescriptions, sedative and anticholinergic load, and drug–drug interactions were evaluated. Of the 116 older patients analyzed, 59 patients were cognitively impaired, and 57 were cognitively normal with an MMSE value > 24. Fifty-nine patients (28 CN, 31 CI) were assisted by the hospital health staff after training.

**Results:**

Participants presented a widespread polypharmacy. Cognitively impaired patients received more prescriptions, more inappropriate prescriptions, had a greater sedative load, and were exposed to more interactions. Staff training had no effect on the prescription pattern.

**Conclusion:**

The results suggest that hospitalized cognitively impaired patients are overprescribed psychotropic drugs and have an excessive sedative and anticholinergic load. Interventions designed to improve dementia care practices in health staff that are not also designed to manage drug polypharmacy do not modify prescription patterns.

## 1 Introduction

The presence of co-morbid chronic illnesses and disability makes elderly patients more vulnerable to adverse events and adverse drug reactions during hospitalization, lengthening their stay in the hospital (Marengoni et al., 2013; Dupouy et al., 2013). Indeed, acute-care hospitalization carries for elderly patients the threat of decreased functional independence, indicating the importance of the identification of at-risk older adults to prevent such a negative outcome (Loyd et al., 2020).

Moreover, the healthcare staff is not always prepared to deal with patients when comorbidity is associated with cognitive impairment and with the presence of behavioral disturbances such as agitation and delirium, conditions frequently not recognized and not properly managed (Morandi and Bellelli, 2020; Lauretani et al., 2020). Indeed, most hospital health staff do not have sufficient psychogeriatric skills despite the great proportion of hospitalized elderly patients with psychiatric disturbances (Wald et al., 2006; Reynolds et al., 2022).

From a general point of view, healthcare staff who care for aged adults are challenged by an overflood of patients with dementia, but only a small minority of seniors are being screened for it (Ingelfinger, 2020). Therefore, it is important to use the available resources to develop person-centered, dementia-friendly care. In the Italian Dementia Friendly Hospital Project, we recorded a series of clinical parameters reflecting the health status and the drug prescriptions during hospital stays before and after a brief (5 h frontal teaching) staff training (Rosi et al., 2023), focusing on improving the management of elderly patients, in particular those with cognitive impairment. The results (Allegri et al., 2022) suggested that the training could improve outcomes for hospitalized older adults with cognitive impairment. In the present article, we report another aspect of hospital care in an extended series of patients, that is, the drug prescription attitude toward over 65 years old patients. Moreover, in the present study, the research on drug prescriptions was extended to the prescriptions at admission and at discharge.

## 2 Methods

### 2.1 Study design and population

Patients aged 65 and older admitted to the General Medicine, Surgery, Cardiology, and Orthopedics Units of the Civic Hospital of Vigevano (Italy) between 1 October 2018 and 31 August 2019 were identified daily from the hospital admission list. Study participants were enrolled within 48 h of admission. Inclusion criteria were patients aged 65 and older with a Mini-Mental State Examination (MMSE) score equal to or higher than 16. Individuals unable to speak Italian or with an MMSE score lower than 16 were excluded because they were considered unable to provide informed consent (Del Signore et al., 2023).

The study was approved (protocol number P-20180060958) by the Ethics Committee of IRCCS Policlinico San Matteo, Pavia, Italy. Written informed consent was obtained from all participants. Participants with an MMSE score ≥16 but ≤24 were considered cognitively impaired. The study population was recruited using a matching-group design to match subjects of the control group with those of the intervention group in terms of age, gender, comorbidity, and cognitive and functional status. We recruited a control group of older adults before implementing the health staff training and an intervention group of older adults after the health staff training. In both the control and intervention groups, we divided the sample into older adults with no cognitive impairment (cognitively normal, CN) and older adults with cognitive impairment (cognitively impaired, CI). After matching, the recruited sample was composed of a total of 116 older patients allocated in four groups, as detailed in Table 1, which shows the demographic characteristics of the sample.

**TABLE 1:** Demographics of the study population.

Recruitment and data collection	Before training the health staff		After training the health staff		*F* (3,112)	*p-value*
Original subgroups	Cognitively normal (n = 29)	Cognitively impaired (n = 28)	Cognitively normal (n = 28)	Cognitively impaired (n = 31)		
Age	79.2 ± 5.8	82.5 ± 7.1	80.0 ± 6.46	81.6 ± 6.6	1.56	0.203
Females, n (%)^a^	13 (44.8)	15 (53.6)	13 (46.4)	18 (58.1)	1.37	0.713
Years of education	7.3 ± 4.0	5.5 ± 2.3	6.7 ± 2.8	5.4 ± 2.8	2.73	0.047
CCI	6.5 ± 2.5	7.3 ± 2.2	6.2 ± 1.8	7.2 ± 1.9	1.73	0.165
MMSE score	26.4 ± 1.6	20.3 ± 2.5*	26.4 ± 1.6	20.1 ± 2.4*	86.23	<.001
Merged subgroups	Cognitively normal patients (57)		Cognitively impaired patients (59)		F(1,114)	*p-value*
Age	79.6 ± 6.1		82.05 ± 6.78		4.22	0.042
Females, n (%)^a^	26 (45.6)		33 (55.9)		1.23	0.266
Years of education	7.0 ± 3.3		5.5 ± 2.5*		7.78	0.006
CCI	6.3 ± 2.2		7.2 ± 2.1*		4.85	0.030
MMSE score	26.4 ± 1.6		20.2 ± 2.5*		262.73	<.001

Note: CCI, Charlson comorbidity index; MMSE, Mini-Mental State Examination. If not otherwise indicated, values are reported as means ± SD; *p ≤ 0.05 compared to the cognitively normal group.

^a^chi-square test value is reported.

As detailed elsewhere (Allegri et al., 2022), participants were evaluated within 48 h of admission and at discharge with the MMSE Barthel Index and Hospital Anxiety and Depression Scale by a team of neuropsychologists. The MMSE was assessed as adapted and normed for age and education in the Italian population (Measso et al., 1993) (range 0–30; normal range >24).

We collected the following information for each subject: current and remote pathological history, diagnosis of dementia prior to and/or during the hospitalization, delirium, and behavioral disorders. In addition, we calculated the Charlson comorbidity index for each subject. Finally, we collected 1) the prescriptions registered at hospital admission from the medical records and an interview of the patients or of their proxy routinely performed by the hospital staff; 2) all the prescriptions for each patient during the hospital stay that were registered every day according to the usual practice; 3) the prescriptions recommended on the discharge form. While the data on the clinical outcome of the patients followed by the trained healthcare staff (Allegri et al., 2022) and the analysis of the training information persistence (Rosi et al., 2023) have already been published, the data on the prescriptions at admission, during the stay in hospital and at discharge, originally collected as hand/computer written documents, were transcribed on an MS Excel file (during 2023) and analyzed starting on September 2023.

Because a preliminary analysis found no differences in the prescription rate/quality between the groups of patients followed by trained *versus* untrained hospital staff, the patients were divided into two groups based solely on their cognitive status (Table 1).

### 2.2 Health staff training

Health staff training has been described in detail elsewhere (Rosi et al., 2023; Allegri et al., 2022). In brief, the training consisted of a 5-h course. Four 1-hour modules were focused on clinical aspects (detection of signs and symptoms of cognitive impairment, management of behavioral and psychological symptoms of dementia (BPSD) and delirium, interaction with patients, and recognition of specific needs). One module was focused on the impact of drug treatments on patients’ cognitive and functional status.

### 2.3 Drug prescription analysis

Drugs were classified according to the Anatomical Therapeutic Chemical (ATC) code, and data were collected in an *ad hoc* created database. Prescriptions were analyzed for appropriateness, sedative load, anticholinergic load, and potential interactions.

#### 2.3.1 Number of drugs/day and appropriateness of the prescription

Patients received various drugs during their hospital stay, which in both number and identity during the whole period of stay. The varied mean number of drugs prescribed every day for each patient was calculated from the medical records, extracting the number of days that each drug was administered and then correcting for the total length of stay according to formula (1):

$$\left[\left(\text{days of }{\text{treatment}}_{\text{drug}\left[1\right]}+\ldots +\text{days of }{\text{treatment}}_{\text{drug}\left[n\right]}\right)/\text{days of total length of stay}\right].$$ (1)

Minimum and maximum values were calculated for each patient, considering the minimum and maximum number of drugs contemporaneously delivered in a single day. In the case of drug prescriptions at admission and at discharge, it was assumed that all the drugs indicated were administered daily unless otherwise indicated in the form.

The appropriateness of the prescription was analyzed using the Beers criteria (American Geriatrics Society Beers Criteria^®^ Update Expert Panel, 2023). In the case of molecules available on the Italian market but not included in the American Geriatrics Society list of medications, the drug was evaluated (i.e., in terms of class, pharmacokinetics) and classified by the team of pharmacologists (S. P., C. L., S.G. among the authors). When applying the Beers criteria, specific care was taken regarding the diagnosis of the patient and the dose prescribed.

#### 2.3.2 Laxatives, intravenous drips, and food supplements

Laxatives, such as senna extract, lactulose, mannitol, and Movicol, were included in the list of prescriptions if used on a regular basis.

Intravenous glucose, sodium, and potassium chloride used to maintain hydration and balance the patient after a medical procedure or on an as-needed basis were not included in the prescriptions; generic multivitamin preparations or other food supplements were also not included.

#### 2.3.3 PPI and prokinetics

PPI and prokinetics prescriptions were considered appropriate if used in patients at risk for ulcers, and low doses of PPIs for patients treated with aspirin for cardiovascular secondary prevention were also considered appropriate. Metoclopramide and alizapride were considered inappropriate, while domperidone was accepted due to the poor blood–brain barrier crossing.

#### 2.3.4 Cardiovascular drugs

While a flecainide prescription is considered inappropriate in the Beers criteria, we considered it appropriate because the prescriptions were written by a cardiologist to control arrhythmia episodes in monitored patients. ACE inhibitors were considered appropriate after checking the potential interactions with other drugs active on the renin–angiotensin–aldosterone system (RAAS) and with potassium-sparing diuretics. Doxazosin was considered appropriate only if prescribed to male patients.

#### 2.3.5 Psychotropic drugs

Haloperidol, as well as other neuroleptics, were considered inappropriate. Benzodiazepines were considered inappropriate unless a specific motivation was present.

### 2.4 Sedative load (SL) definition

SL was calculated for each subject according to a published model (Linjakumpu et al., 2003). Drugs were rated from 0 to 2, with level 0 indicating no sedative activity (Groups 3 and 4) and level 2 (Group 1) indicating an elevated sedative activity. The sedative load scores relative to each drug simultaneously taken by the patient were summed up to obtain the total score.

### 2.5 Anticholinergic load (AL) definition

The AL was calculated according to the scoring system of the Anticholinergic Drug Scale (Carnahan et al., 2006). Drugs are rated from 0 to 3, with level 0 indicating no anticholinergic activity and level 3 indicating an elevated anticholinergic activity. The anticholinergic scores relative to each drug simultaneously taken by the patient were summed up to obtain the total score.

### 2.6 Drug interactions

Drug interactions were evaluated using the Drug Interactions Checker program (www.drugs.com/drug_interactions.html) and, if needed, were further checked by the pharmacologist team.

### 2.7 Statistical analyses

Mean and standard deviation were calculated for each variable.

We first performed a series of one-way ANOVAs to evaluate the equivalence of the intervention and control groups on some demographic variables (age and years of education) and some baseline characteristics at admission (MMSE and CCI). The chi-square test was used to evaluate the gender equivalence across groups. To evaluate the effect of the dementia-friendly intervention, a repeated-measures analysis of variance (ANOVA) 2 (cognitive status: CI vs. CN) X 2 (groups: intervention vs. control) was conducted on the mean number of prescribed drugs/day. Once the lack of effect of the training intervention on the prescription rate was observed, the following analyses were done on the two groups divided based on the cognitive status. To test interactions, *post hoc* pairwise comparisons were performed using Student’s t-test following one-way ANOVA, considering *p* < .05 as the threshold of statistical significance. The chi-square test was used to evaluate the frequency of N-class drug use. A bivariate analysis based on Pearson’s r correlation was used to explore the relationship between some of the considered characteristics.

## 3 Results

The cognitively normal and cognitively impaired subgroups presented a significant difference in the MMSE values, as per the protocol, and in years of education, as has been frequently observed in groups of CI subjects. In addition, the number of comorbidities was higher (t (114) = 2.020, *p* = 0.03) in cognitively impaired subjects than in CN subjects.

Participants presented widespread polypharmacy, receiving, on average (whole sample), more than six drugs/day (not shown). Both the mean number of drugs prescribed/day and the maximum number of drugs received per single day of hospitalization were significantly higher in the cognitively impaired patients than in the cognitively normal subjects (Table 2). The evaluation of the maximum number of drugs taken in a day was based on the daily hospital records. The average number of daily drug prescriptions was similar throughout the groups at admission and at discharge.

**TABLE 2:** Mean number of drugs/day (and minimum–maximum range during hospitalization).

Group (N. of subjects)		Cognitively normal patients (57)	Cognitively impaired patients (59)
Mean number of drugs/day	B	6.0 ± 3.2	6.5 ± 3.2
	H	6.0 ± 2.9	7.5 ± 4.9*
	D	6.7 ± 2.7	6.8 ± 3.0
Mean minimum – Maximum number of drugs/day	H	Cognitively normal patients (57)	Cognitively impaired patients (59)
		3.3 ± 1.8 7.7 ± 2.6	3.3 ± 2.2 8.9 ± 3.3^

B: prescriptions before admission; H: prescriptions during hospitalization; D: prescriptions at discharge from the hospital.

If not otherwise indicated, values are reported as means ± SD; *p = 0.05 (t = 1.98, df = 114) compared to cognitively normal; ^ *p* = 0.036 (t = 2.12, df = 114) compared to cognitively normal.

### 3.1 Correlations between drug use, age, and comorbidity

When working on the data obtained on the use of drugs before hospital admission, age did not correlate with the number of prescriptions either in the whole sample (r = −0.016; *p* = 0.86) or in the subgroups within the age range of the investigated patients (66–94 years). Not surprisingly, the subjects who had a higher number of comorbidities in addition to the illness for which they were hospitalized received more prescriptions (whole sample, r = 0.45; *p* = 0.001), and this was also true when examining CN (r = 0.51, *p* = 0.001) and CI (r = 0.38, *p* = 0.003) patients separately.

Similar results were obtained by examining the data during the period of stay in the hospital. The subjects who had a higher number of comorbidities received more prescriptions [whole group: r = 0.36; *p* < 0.001; CN: r = 0.44, *p* = 0.001; CI: r = 0.28, *p* = 0.0028]. The general picture did not change when examining the data at discharge from the hospital.

### 3.2 Most prescribed drugs

From a general point of view, the first three ATC classes (A, alimentary tract and metabolism; B, blood and blood-forming organs; and C, cardiovascular system) were the most represented, totaling altogether between 63.5% and 78.9% of all prescriptions. Among the other ATC classes, the N class was the next most prescribed and was more represented in the cognitively impaired patients in all three periods (at admission, during the stay in hospital, and at discharge), as detailed further.

As far as the use of individual drugs, among the prevalent ones, furosemide was prescribed to more than half of the patients (56.9%); enoxaparin (37.0%), acetaminophen (31.0%), and bisoprolol (30.2%) were also very frequently prescribed (around one in every three patients); followed by acetylsalicylic acid (22.4%), mainly used for secondary prevention following cardiovascular and cerebrovascular events. Proton pump inhibitors were the champions; indeed, the number of prescriptions as a class was 104, corresponding to 89.7% of all patients.

### 3.3 Use of psychotropic drugs

The number of prescriptions of psychotropic drugs in cognitively impaired patients was almost double that given to cognitively intact patients in all three periods of observation, as shown in Table 3.

**TABLE 3:** Prescriptions of psychotropic drugs at admission, during hospitalization, and at discharge.

Cognitive status	Total number of patients	Number of N-class prescriptions	Number of patients receiving at least one N-class prescription (percentage)	Number of N-class prescriptions	Number of patients receiving at least one N-class prescription (percentage)	Number of N-class prescriptions	Number of patients receiving at least one N-class prescription (percentage)
		Prescriptions before admission		Prescriptions during hospitalization		Prescriptions at discharge	
Normal	57	18	13 (22.8)	34	22 (38.5)	19	13 (22.8)
Impaired	59	50	29* (49.1)	69	38^ (64.4)	51	30° (50.8)

Comparisons with the respective cognitively normal group: *p = 0.003 (Χ^2^ = 8.71, df = 1); ^*p* = 0.005 (Χ^2^ = 7.73, df = 1); °*p* = 0.02 (Χ^2^ = 9.72 df = 1).

During the hospital stay, the number of N-class drugs prescribed to the patients was higher than at admission or at discharge, both in cognitively normal and cognitively impaired patients.

As shown in Table 3, CI patients received altogether twice as many prescriptions of drugs belonging to the ATC group N, used as hypnotics, anxiolytics, antidepressants, antipsychotics, antiepileptics, and opioid analgesics. Among psychotropic drugs, benzodiazepines as a class held the highest ranking (30.5% of the listed prescriptions in the CI group).

During the hospital stay, the use of psychotropic drugs was higher than the admission values (both as number of prescriptions and number of patients receiving at least 1 N-class drug prescription) both in CI and in CN patients (with increments in the number of prescribed N-class drugs ranging from +38% to +89%). At discharge, the use of psychotropic drugs returned to values comparable to those observed at admission in the respective group.

The numbers of drugs given to control delirium or agitated behavior (as indicated by treatment with an antipsychotic or a benzodiazepine not lasting more than 2 days) were too small for a statistical comparison; however, it should be noted that CN patients received three such prescriptions altogether while CI patients received six.

### 3.4 Sedative and anticholinergic load

CN patients had a lower sedative load (SL) than CI patients (Table 4). During the hospital stay, the CI patients had a significantly higher mean SL value, and the maximum SL reached a very high value of 10. Moreover, during the inpatient period, the average calculated SL values were higher both in CN and CI patients than the values at admission and at discharge (see Table 4).

**TABLE 4:** Sedative and anticholinergic load.

Score	Sedative load score						Anticholinergic load score					
	Cognitively normal patients (57)			Cognitively impaired patients (59)			Cognitively normal patients (57)			Cognitively impaired patients (59)		
	B	H	D	B	H	D	B	H	D	B	H	D
Mean (±SD)	0.82 ± 1.24	1.30 ± 1.71	0.49 ± 0.98	1.39 ± 1.62^a^	2.22 ± 2.15^b^	1.25 ± 1.79^c^	0.67 ± 0.79	1.26 ± 1.08	1.07 ± 1.00	1.16 ± 1.53^d^	2.00 ± 1.95^e^	1.32 ± 1.75^f^
Min-Max	0–5	0–6	0–4	0–6	0–10	0–9	0–4	0–5	0–4	0–8	0–8	0–8
Patients with at least one load (%)	20 (35.1)	35 (61.4)	14 (24.6)	33 (55.9)^g^	46 (78)^h^	31 (52.5)^i^	30 (52.6)	43 (75.4)	38 (66.7)	35 (59.3)^j^	46 (78.0)^k^	33 (55.9)^l^

B: prescriptions before admission; H: prescriptions during hospitalization; D: prescriptions at discharge from the hospital.

^a^ t = 2.10; *p* = 0.037 compared to the respective cognitively normal group (B) cognitively normal.

^b^ t = 2.55; *p* = 0.012 compared to the respective cognitively normal group (H).

^c^ t = 2.83; *p* = 0.005 compared to the respective cognitively normal group (D).

^d^ t = 2.21; *p* = 0.029 compared to the respective cognitively normal group (B).

^e^ t = 2.62; *p* = 0.010 compared to the respective cognitively normal group (H).

^f^ t = 0.94; *p* = 0.340 compared to the respective cognitively normal group (D).

^g^ *p* = 0.038 (Χ2 = 4.30, df = 1) compared to the respective cognitively normal group (B).

^h^ *p* = 0.052 (Χ2 = 3.77, df = 1) compared to the respective cognitively normal group (H).

^i^ *p* = 0.002 (Χ2 = 9.56, df = 1) compared to the respective cognitively normal group (D).

^j^ *p* = 0.468 (Χ2 = 5.27, df = 1) compared to the respective cognitively normal group (B).

^k^ *p* = 0.747 (Χ2 = 0.10, df = 1) compared to the respective cognitively normal group (H).

^l^ *p* = 0.236 (Χ2 = 1.41, df = 1) compared to the respective cognitively normal group (D).

Considering the anticholinergic load, the table shows that CN patients had a lower mean value than CI patients at admission and during the hospital stay period. The trend was present but not significant at discharge. The average anticholinergic load was higher during the hospital stay than at the admission level. In addition, the anticholinergic load at discharge trended toward higher values than at admission. When considering the number of patients with at least one SL or AL, a significant difference was observed in the case of the cognitively impaired patients for SL at admission and at discharge, but it did not reach the significance threshold during the hospital stay and not in the case of AL in any of the three periods. This observation suggests that the number of sedative and anticholinergic drugs/patient was the major contribution to the difference rather than the number of patients treated.

Notably, no prescriptions of drugs belonging to the antidementia or to the nootropic class were found in the whole sample, although half of the sample had cognitive problems. In addition, prescriptions for cerebrovascular drugs were almost absent.

### 3.5 Potentially inappropriate prescriptions (PIPs)

The CI group (who presented a higher number of prescriptions of psychotropic drugs) than the CN patients had, during their hospital stay, more PIPs, calculated as percentage of prescriptions (18.6 vs. 11.4), mean PIPs per patient (2.02 vs. 1.07 *p* = 0.001), and numbers of patients presenting at least one (52 vs. 35) and more than three PIPs (16 vs. 6). It should be noted that psychotropic drug prescriptions were not the only inappropriate prescriptions. As expected, a positive correlation was observed examining the whole sample between the number of prescriptions and the PIPs (as an example: Pearson’s r = 0.54, *p* < 0.001 during the inpatient period).

### 3.6 Drug–drug interactions and interactions with food

Several interactions of potential clinical interest were observed. The absolute number of major interactions was higher in all three periods of observation in CI patients (B: 25; H:86; D:47) than in CN patients (B: 11; H:64; D:28). The combined number of interactions was (major + moderate + minor) higher during the hospital stay than at admission in both CN (+87%) and CI (+150%) patients. The average number of interactions/patient in the whole sample was 6.3 at admission, 11.9 during the hospital stay, and 7.9 at discharge. The percentage of major interactions ranged from 3.5% (CN at admission) to 10.0% (CI during hospital stay). Finally, several food/drug interactions were present (ranging from 147 to 190), and therapeutic duplications peaked during the hospital stay period (127, considering both CI and CN patients). However, none of these differences, when calculated as mean interactions/patient, was significant when comparing CN and CI patients. As expected, a positive correlation for the whole sample was observed between the number of prescriptions and the interactions (as an example: major interactions: r = 0.34, *p* = 0.001; moderate interactions r = 0.65, *p* < 0.001; minor interactions: r = 0.31, *p* < 0.001 during the inpatient period).

## 4 Discussion

The most relevant results are that CI patients received more psychotropic drugs and had higher sedative and anticholinergic loads. From a general point of view, the data are in line with those reported at the population level by Christensen et al. (2019) in older adults (>60 years).

In terms of the frequency of use of specific drug classes, we observed mostly alimentary tract and metabolism, blood and blood-forming organs, and cardiovascular drugs without significant differences between groups. PPIs held the number one rank in both groups, as was also noticed in a previous study (Allegri et al., 2017).

In addition, we observed that psychotropic drugs had a higher prevalence in the CI patients in all three periods considered, that is, at admission, during the hospital stay, and at discharge. The overuse of these drugs in the CI patients was evident during the hospital stay; 22/57 (39%) of the CN patients and 38/59 (64%) of CI patients received them (see Table 3 for the other periods). The data are in line with the concept that cognitive impairment represents the main risk factor for psychotropic drug use in hospitalized older patients and the frequent association of neuroleptic drugs when a dementia diagnosis is present (Arnold et al., 2017). Special attention to the possibility of deprescribing is required by physicians providing post-acute care (American Geriatrics Society Beers Criteria^®^ Update Expert Panel, 2023; Conti et al., 2021; Rubin, 2023). We observed that at least in some cases (consulting the daily logs), the use of neuroleptics was limited to 1–2 days. However, in several other cases, the treatment was prolonged.

It should be noted that during the hospital stay, psychotropic drugs were more represented in the cognitively impaired patients irrespective of the training of the hospital staff (not shown), indicating that the training had favorably modified other patient and staff parameters (Allegri et al., 2022; Rosi et al., 2023) but did not affect the prescription attitude.

In line with the above observations, the average sedative load (Table 4) of CI patients was higher than that in CN patients in all the periods of observation (averaging from +77% to +155%). The anticholinergic load was significantly higher in CI patients at admission and during hospital stay (respectively +73% and +58%), while it was not statistically different at discharge. As in the case of the sedative load, the anticholinergic load was higher during the hospital stay than at admission.

The use of drugs with anticholinergic properties in elderly patients was found to be associated with cognitive and functional decline (Brombo et al., 2018). Lattanzio et al. (2018) reported data on a sample of 817 geriatric patients, warning that an anticholinergic burden (ACB) at discharge has negative prognostic implications. This concept of the negative impact of ACB is further stressed by data (D'Alia et al., 2020) showing that the ACB score at discharge may predict mortality among older patients with low hand grip strength. Moreover, Pasina et al. (2020) suggested that in the case of several drugs (e.g., quetiapine, olanzapine, paroxetine, and promazine) for which alternatives are available, the therapy should be optimized, and the anticholinergic burden decreased. An article on more than 19,000 initially healthy older people warned of the increased risk of incident dementia and stroke associated with anticholinergic exposure (Lockery et al., 2021), strengthening once more the importance of actively controlling the ACB in elderly patients. In a study on anticholinergic drug use and risk of mortality, specifically for people with dementia (based on an observation of 25,418 patients in Northern Ireland), McMichael et al. (2021) found that a higher anticholinergic burden was associated with significantly higher mortality rates, further stressing the importance of reducing the anticholinergic burden. The negative impact of anticholinergic medications has been recently further underscored by Smith and Fligelstone (2024) and also by Rodríguez-Ramallo et al. (2024), who suggested guidance for deprescription of anticholinergic/sedative medications.

In light of these considerations, the fact that mean cholinergic loads at discharge are greater than those at admission suggests the need for action.

Higher scores on the Drug Burden Index (that recapitulates the sedative and anticholinergic load) are associated with worse clinical outcomes and functional impairment in various domains, including cognition (Liu et al., 2024).

We did not find any prescription of nootropics and antidementia drugs. On the other hand, of the 59 patients classified as CI, only six (10.0%) had a diagnosis of dementia or were classified as CI prior to or during the hospitalization, indicating that in 90% of the cases, this is a neglected and untreated condition. The undertreatment of cognitive impairment is consistent with a previous study (Allegri et al., 2017) on the non-hospitalized population residing in Vigevano. Literature data (Garcia et al., 2023) suggest that in other contexts, the percentage of patients with untreated mild AD ranges from 9% to 26%, which is a much lower proportion. An overview of the Italian situation based on administrative data is offered by Ippoliti et al. (2023). The data comparing the AD prevalence with the prevalence treatment use show a gap that becomes larger as age increases, suggesting undertreatment levels around and higher than 60% for >75-year-old patients. The importance of diagnosing dementia in older people in the general hospital is also underscored at the international level (Sommerlad et al., 2019). The undertreatment of dementia contrasts with recent literature, underscoring the value of the approved treatments (Zuin et al., 2022; Lombardi et al., 2022). It should also be stressed that a Japanese study (Suzuki et al., 2020) has shown that the use of antidementia drugs reduces the risk of potentially inappropriate medication use, in particular, psychotropic drugs. Therefore, the lack of treatments for cognitive deficits may also impact other health-related parameters.

The combined effects of a greater number of prescriptions and an increased sedative and anticholinergic load contributed to the higher frequencies of PIPs in CI patients than in CN patients. The frequency of patients presenting at least one PIP was higher in the CI group during the stay in hospital (CI: 52/59 (88%) vs. CN: 35/57 (61%) patients). In the other periods, the values in the CI group were higher than in CN patients, but the difference did not reach statistical significance. In general, the number of PIPs, as well as the number of drug–drug and drug–food interactions, was correlated to the number of prescriptions. PIP frequency was not different from our previous study on nursing home patients (Allegri et al., 2017) and in line with the literature, although higher values were recorded. On the other hand, the published data reveal a certain degree of heterogeneity that may be explained by several factors, including the specific criteria of appropriateness and updates used over time, the setting (community, hospital, nursing home), and the inclusion criteria (age, comorbidity, polypharmacy). In particular, Onder et al. (2003), using the 2003 Beers criteria, reported that 28.6% of 5,142 patients received at least one or more inappropriate drugs and 5.6% received two or more during their hospital stay. Those values are definitely lower than the ones we observed in our whole sample of patients (61.4% for one or more PIPS and 10.5% for three or more in hospitalized CN patients).

In another study on six European hospitals with a total of 900 patients, the percentage of patients receiving at least one PIP ranged from 22.7% to 77.3% according to the site and the criteria adopted (Corsonello et al., 2009). In the cited study, the data on PIPs using the 2003 Beers criteria ranged from 22.7% to 43.3%, depending on the hospital considered.

Jungo et al. (2021) recently studied the utilization of potentially inappropriate medications (PIM) in older adults using electronic health records from seven hospitals/medical centers in Massachusetts (2007–2014, a very large database of hundreds of thousands of patients). Participants were older than 65 years and presented multimorbidity and polypharmacy. Using the American Geriatrics Society 2019 version of the Beers criteria, the authors found that more than 69% of the patients assumed at least one PIM, a percentage near the one we observed in our sample of patients with similar characteristics. The role and frequency of PIPs are also underscored in a recent investigation on nearly three million Danish hospital patients, pointing to the association with adverse outcomes (Rodríguez et al., 2022).

Our study was done on a small cohort, although the selected sample and relatively small numbers may represent a significant proportion of the patients. On the other hand, the comparison between CI and CN patients balanced most of the confounding variables in our study, suggesting that the differences between the two groups were real and associated with cognitive impairment. Notably, in our experience, the number of PIPs increased during the hospital stay in both CI and CN patients, likely due to the increased therapy intensity, and was like baseline at discharge (with a trend toward increased values), indicating that the hospital did not put in action interventions meant to reduce PIM in the elderly patients.

While some of the determinants of PIPs are emerging (age, comorbidity, number of prescriptions, cognitive status, and socioeconomic status), it is still uncertain what the benefits of reducing them will be. Indeed, Pazan et al. (2021), examining 1998–2019 articles on the relationship between drugs and frailty, commented that the data are inconclusive and that randomized clinical trials on this topic are needed. Notably, within this context, Ie et al. (2020) designed a protocol to determine whether medication optimization in elderly inpatients can improve clinical outcomes. Within this context, Mangin et al. (2018) published a position statement and 10 recommendations to reduce inappropriate medication use and polypharmacy. On the other hand, a trial using a computerized decision support tool for a comprehensive drug review of older people with polypharmacy showed a limited impact (Rieckert et al., 2020), indicating that more experience is needed and that a patient-tailored perspective and teamwork may be important.

Studies on improving the quality of medication use and medication safety are important priorities for prescribers, as well as non-drug-based approaches for behavioral disturbances. In the case of antipsychotic use, it has been shown that psychosocial interventions may lead to a substantial reduction in psychotropic drug prescription (Birkenhäger-Gillesse et al., 2018). Moreover, medication reconciliation and medication review procedures indicate that interventions should be focused and tailored, creating a consensus on drug therapy involving health personnel and the patient (Beuscart et al., 2021; Crowley et al., 2020). Preventive training may be helpful and make the health staff more prone to accept the discussion of drug therapy, but teamwork remains an important goal. In addition to during inpatient care, the risk of polypharmacy and potentially inappropriate drugs is particularly present in residential care patients and is emerging, for example, from the Pharmacovigilance in the Elderly study (PharE study), which offers suggestions for avoiding prescription mistakes prescriptions in the elderly and points to the lack of commonly shared guidelines to adequately cope with the treatment complexity of comorbidities in the elderly (Gareri et al., 2021), a problem worsened by cognitive impairment.

Altogether, the results suggest that CI patients present a pattern of drug prescription that differs from CN subjects. CI patients received more prescriptions, more inappropriate prescriptions, and had a greater sedative and anticholinergic load. This series of negative patterns suggests that CI patients are at risk of negative outcomes of their hospital stay that are partially due to the contribution of inappropriate drug use. Accordingly, a very practical suggestion is that the evaluation of the cognitive status and the detection of cognitive impairment of a patient at admittance could trigger a series of actions, including greater attention to prescriptions, that might improve patient outcomes. Several studies support this statement, including case reports and clinical studies. In an interesting case report, Gareri et al. (2020) underscore the importance of the collaboration between geriatricians and pharmacologists to provide a wide prescription and deprescription process for elderly patients with attention to the use of excessive sedation and the possibility of sustaining memory function through the cholinergic approach. The importance of checking the drugs taken by patients affected by dementia and deprescribing is also underscored in a recent analysis of the medications prescribed for 205 older outpatients; their prescriptions were periodically examined, and drugs with the lowest benefit-to-harm ratio and probability of adverse withdrawal reactions were deprescribed (Gareri et al., 2024).

In addition, the results suggest that an intervention focused on improving dementia care practices in health staff that has the potential to improve outcomes for hospitalized older adults with cognitive impairment but was not directly designed to manage drug polypharmacy is not sufficient to modify drug prescription patterns. The latter action requires more specific training directed toward improving drug prescription patterns targeting prescribing staff. Such a training program should be designed, and the specific outcomes tested. Moreover, initiatives such as the REPOSI register (Mannucci et al., 2018) may help to detect polypharmacy, and medication review procedures may be a more appropriate tool for intervention after detecting the presence of an inappropriate prescription in a certain health setting/structure.

## Data Availability

The raw data supporting the conclusions of this article will be made available by the authors, without undue reservation.
